# Sex-specific effects of prenatal methamphetamine exposure on activity and blood pressure in aged offspring

**DOI:** 10.3389/fnbeh.2026.1772937

**Published:** 2026-07-13

**Authors:** A’Kaychia T. Lowery, Isaac G. Johnson, Anna G. Mitchell, Daniela Rüedi-Bettschen

**Affiliations:** 1 Graduate Program in Neuroscience, University of Mississippi Medical Center, Jackson, MS, United States; 2 Department of Psychiatry and Human Behavior, University of Mississippi Medical Center, Jackson, MS, United States

**Keywords:** blood pressure, locomotor activity, long-term effects, methamphetamine, prenatal exposure

## Abstract

**Introduction:**

Methamphetamine use during pregnancy is a global health issue, and there is limited knowledge on the cardiovascular and behavioral effects of prenatal methamphetamine exposure in adults. This study investigated how prenatal methamphetamine exposure in rats, via maternal intravenous self-administration, affected methamphetamine sensitivity and cardiovascular function in aged offspring compared to controls.

**Methods:**

The aged offspring were generated from dams either self-administering methamphetamine daily under extended-access conditions before and throughout pregnancy, or their respective sham control dams. Spontaneous and methamphetamine-induced locomotor activity was assessed in male and female offspring of both exposure groups after a range of methamphetamine doses at age 15–17 months. Cardiovascular function was assessed in male and female offspring aged 18–20 months of both groups.

**Results:**

Baseline blood pressure and angiotensin II (AngII)-induced hypertension were determined non-invasively via tail cuff measurements. Methamphetamine dose-dependently increased locomotor activity compared to baseline activity in both sexes and both exposure groups. In both sexes, AngII did not increase diastolic or systolic blood pressure in methamphetamine-exposed offspring, yet induced hypertension in controls. Methamphetamine administration induced dose-dependent hyperactivity in both exposure groups, with methamphetamine-exposed offspring showing resistance to stereotypic effects and thus increased activity at 3.2 mg/kg compared to controls. Notably, methamphetamine-exposed female offspring had a tendency to hypertension at baseline, driven by higher diastolic blood pressure, and showed reduced hyperactivity compared to controls at 1.0 mg/kg. Males did not exhibit these changes.

**Conclusion:**

These findings suggest that aged female offspring are particularly sensitive to the impacts of prenatal methamphetamine exposure on blood pressure and methamphetamine sensitivity.

## 1 Introduction

Methamphetamine (METH) is a commonly used psychostimulant. A large proportion of METH users are young adults. METH use has been shown to impair decision-making and increase the pleasurable effects of sex, thus unintended pregnancies are common (Rawson et al., 2002; Kalechstein et al., 2003; Arria et al., 2006; Zapata et al., 2008; Berry et al., 2022; Lorvick et al., 2012). The negative effects of METH use are well described in adults, which include effects on the central nervous, reproductive, and cardiovascular systems, including hypertension, tachycardia, myocardial infarctions, hyperthermia, altered reproductive hormones, and neurotoxicity (Albertson et al., 1999; Bhatia et al., 2022; Busceti et al., 2024; Chen et al., 2024). In addition, the physiological and behavioral effects of METH use exhibit sex-specific differences (Daiwile et al., 2022). In clinical studies, METH users present with a higher prevalence of severe hypertension, pulmonary arterial hypertension, and hemorrhagic stroke compared to controls (Jones and Rayner, 2015; Lappin et al., 2017; Lv et al., 2016; Pike et al., 2014; Zamanian et al., 2018), and cardiovascular disease is highly prevalent in METH-associated deaths (Darke et al., 2017). However, much less is known regarding the effects of METH in pregnant women, the developing fetus, and the potential long-term effects of prenatal METH exposure. Only a small number of studies have described METH-induced cardiovascular effects during pregnancy, but these findings indicate that METH use can lead to tachycardia, hypertension, and placental insufficiency during pregnancy (Carter et al., 2016; Good et al., 2010; Gorman et al., 2014; Plessinger, 1998). Clinical studies have identified developmental and behavioral effects in children due to prenatal METH exposure. Cohort studies of infants have identified higher susceptibility to congenital heart defects, delayed developmental milestones, low birth weight, and smaller head circumference (Radgoodarzi et al., 2021; Shah et al., 2012; Wright et al., 2015). Prenatal exposure to METH has also been associated with increased emotional reactivity and physiological stress, a higher incidence of attention-deficit/hyperactivity disorder, and poorer quality of movement in children (LaGasse et al., 2012; Wright et al., 2015; Kirlic et al., 2013; Diaz et al., 2014). However, it is difficult for clinical studies to identify the direct adverse side effects of prenatal METH exposure on offspring due to several confounding factors, such as stressful environments, comorbidities, poly-substance use, and socioeconomic status (Hayer et al., 2024; Kunkler et al., 2022). Preclinical studies have shown that prenatal METH exposure leads to delayed postnatal development, increased anxiety, impaired cognition and memory, and increased sensitivity to drugs of abuse (Slamberová et al., 2005; Bubenikova-Valesova et al., 2009; Hruba et al., 2010; Hrubá et al., 2012; Šlamberová, 2012; McDonnell-Dowling et al., 2014; McDonnell-Dowling and Kelly, 2015, 2016; Fialová et al., 2015; Holubová-Kroupová and Šlamberová, 2021; Li et al., 2025; Rüedi-Bettschen and Platt, 2017; Pareek et al., 2023). Further studies have also shown that prenatal METH exposure increases susceptibility to hepatotoxicity, decreases seizure latencies, and impacts endothelial function (Li et al., 2024; Slamberová and Rokyta, 2005; Harrison et al., 2021). Overall, studies in humans and rodents are uncovering the effects of prenatal METH exposure on susceptibility to behavioral disorders and physiological dysfunctions from infancy to young adulthood. However, the long-term effects extending into advanced age, and to what extent these effects are sex-dependent, are still understudied. In the current study, we utilize our established model (Rüedi-Bettschen and Platt, 2017) of maternal METH self-administration under extended-access conditions to evaluate how prenatal METH exposure affects sensitivity to METH and potential cardiovascular dysfunction in aged offspring, and whether these effects are sex-dependent. In general, we expect that methamphetamine-exposed offspring will be more sensitive to methamphetamine effects in adulthood and that the effects will be sex-specific.

## 2 Materials and methods

### 2.1 Animals and surgery

All experiments were conducted in accordance with the National Institutes of Health (NIH) Guide for the Care and Use of Laboratory Animals and the University of Mississippi Medical Center Animal Care and Use Committee. Adult female and male Sprague-Dawley rats used to generate the study animals were purchased from Charles River Laboratories (Wilmington, MA), approximately 65 days of age, weighing 300–350 g or 200–250 g, respectively, at arrival.

All rats (breeders and study animals) were housed in single-sex pairs in standard shoebox cages in a temperature and humidity-controlled vivarium with a reverse 12-hr light/dark cycle (lights on at 20:00 h). Food and water were available *ad libitum*. Behavioral testing occurred during the dark phase.

#### 2.1.1 Generation of study animals

To generate the METH-exposed and control offspring used for this study, we utilized a maternal METH self-administration model in Sprague-Dawley rats, similar to that described by Rüedi-Bettschen (Rüedi-Bettschen and Platt, 2017). All breeder females were implanted with jugular vein catheters as described below. Half of the females (one per cage) were randomly chosen and trained to self-administer METH (0.05 mg/kg/infusion) under a fixed-ratio (FR) schedule for daily 6-hr sessions. Experimental sessions were conducted in operant chambers (Med Associates, Georgia, VT) equipped with an infusion pump, two levers, a white stimulus light above each lever, and a house light on the opposite wall. An infusion line was connected to a rotating swivel and routed through a spring tether that was attached to the skin button. The recording of responses, activation of lights, and infusion pump were computer-controlled (MED-PC IV, Med Associates, Georgia, VT).

Females were weighed before each session, and the infusion duration was adjusted via the computer program (maximum ± 0.5 s) to ensure an accurate infusion dose. Completion of the FR on the active lever led to a 4-s drug infusion concomitant with the illumination of the stimulus light above the lever, followed by a 6-s time-out during which the light remained illuminated. Lever presses on the inactive lever were recorded, but had no programmed consequences. Initially, rats were trained under an FR1 schedule until they met the criterion of at least 3 days with at least 2 days with over 15 infusions/session; after which, the FR requirement was increased to 2. The remaining females served as controls (sham). They underwent catheterization surgery and were weighed and flushed daily, but remained in the home cage during the daily METH self-administration sessions.

Once stable levels of self-administration were reached (at least 90 infusions/session with no upward or downward trends in the number of infusions over a 3-day period, with the number of infusions within ± 20% of the last 3-day mean), a male was introduced to the home cage of a METH-Sham female pair and allowed to mate. Males remained with the females until the females were close to delivering their litters, as estimated by the time elapsed since the first signs of pregnancy (e.g., obvious weight gain, a rounded abdomen). METH self-administration was continued daily throughout the mating and gestational periods and ceased the day a litter was born.

**FIGURE 1:**
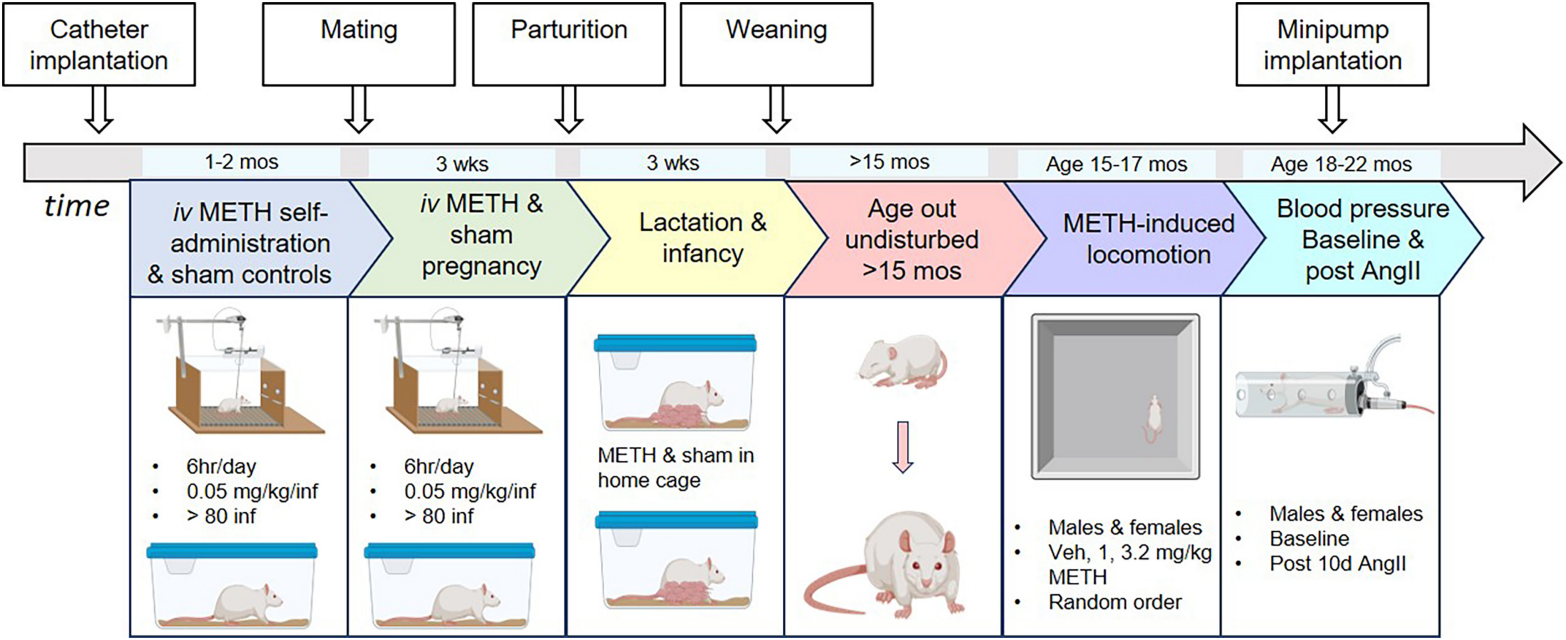
Timeline and order of experimental time points for the generation and testing of the study animals. For each experiment in aged offspring, unrelated offspring were used to avoid confounding litter effects. Males and females, however, were siblings. This image was created with the assistance of BioRender.com.

Generated male and female offspring (either prenatally METH-exposed or sham) were allowed to age until testing at 15–22 months, depending on the test. Female rats had stopped cycling at the time of testing. Figure 1 illustrates the timeline of the offspring generation and timing of tests. For each experiment, unrelated offspring were used to avoid confounding litter effects. Males and females, however, were siblings.

#### 2.1.2 Surgery

Female breeder rats were implanted with indwelling jugular vein catheters (3 Fr, medical-grade polyurethane, Instech Laboratories, Plymouth Meeting, PA) under isoflurane anesthesia. The distal end of the catheter was routed subcutaneously (*s.c.*) and connected to a vascular access button (Instech Laboratories) implanted in the mid-scapular region, permitting access to the catheter without exposing it to air. Catheters were flushed daily with heparinized saline (30 IU/ml) to maintain patency. Animals were allowed to recover from surgery for 7 days before starting METH self-administration.

To administer AngII (50 ng/kg/day) in aged offspring, Alzet osmotic minipumps (Durect Corporation, Cupertino, CA) were implanted *s.c.* posterior to the scapulae.

### 2.2 Drugs

Methamphetamine hydrochloride and AngII were purchased from Sigma Aldrich (St. Louis, MO) and prepared in 0.9% sterile saline solution.

### 2.3 METH-induced locomotor activity

Evaluations of baseline and METH-induced locomotor activity were conducted in beam-break activity chambers (Columbus Instruments, Columbus, OH) located in individual cubicles. Female and male offspring, at 15–17 months of age, were allowed to acclimate to the testing room for 60 min prior to experimental sessions to reduce potential stress from transportation. To assess METH sensitivity during the active cycle, experimental sessions were conducted twice a week during the dark cycle at the same time of day.

Spontaneous locomotor activity was recorded for each male (*n* = 12; 4 METH-exposed, 8 control) and female subject (*n* = 13; 6 METH-exposed, 7 control) during a 30-minute baseline period at the beginning of each experimental session to assess potential changes in baseline activity. Following this baseline assessment, all rats received a *s.c*. injection of either vehicle (saline), 1.0 mg/kg METH, or 3.2 mg/kg METH, with dose order randomized across sessions, in a within-subject, repeated-measures design. These doses were selected based on Pareek et al. (2023), who reported sex-specific behavioral effects at these concentrations in METH-exposed versus control offspring. Immediately after injection, subjects were returned to the activity chambers, and locomotor activity was recorded for an additional 90 min.

### 2.4 Blood pressure measurement

Blood pressure (measurements before and after AngII treatment) was noninvasively collected in real time with the CODA tail cuff apparatus (Kent Scientific Corporation, Torrington, CT). The apparatus consisted of four plastic cylindrical channels that restrained the offspring. Each channel was paired with a Volume Pressing Recording (VPR) that was attached around the base of the subject’s tail. Computer recordings of blood pressure parameters occurred in real time during the dark cycle.

Male and female offspring, at 18–22 months of age, were allowed to acclimate to the testing room for 60 min prior to experimental sessions to reduce potential stress from transportation. Each male (*n* = 9; 4 METH-exposed and 5 control) and female subject (*n* = 18; 8 METH-exposed and 10 control) was habituated to the tail cuff apparatus for at least 2 weeks to ensure potential stress did not interfere with the blood pressure assessment. Baseline blood pressure measurements were then recorded for 2 days. After recording the baseline, osmotic mini pumps containing AngII were implanted in each rat. The pump delivered 50 ng/kg of AngII daily at a controlled rate for 14 consecutive days. This dose constitutes a subpressor dose of AngII, which induces a modest increase in blood pressure after several days of infusion (Dickinson and Lawrence, 1963; Pelaez et al., 2003). To ensure 10 days of AngII exposure at steady state, blood pressure measurements were recorded again via tail cuff on days 12 and 13 post-implantation. Measures were taken in 30-s intervals for a total of 15 data points per session, capturing mean, diastolic, and systolic blood pressure.

### 2.5 Statistical analysis

Spontaneous locomotor activity differed between the two cohorts tested, regardless of prenatal exposure. Therefore, data were normalized as percent change relative to baseline activity for each subject before subsequent testing. Due to the normalization of the locomotor data, sex differences were not analyzed. Each sex was analyzed separately for each dose using a mixed-model repeated-measures (RM) two-way Analysis of Variance (ANOVA) with the within-subject RM factor of time bins and the between-subjects factor of exposure (sham-exposed, METH-exposed).

To analyze blood pressure parameters (i.e., mean, diastolic, and systolic), blood pressure measurements before and after AngII treatment were collected for 2 days, averaged across the 15 trials per day, and used to generate a single data point per rat for baseline and post-AngII. The averages of each blood pressure parameter were analyzed using a mixed-model three-way RM ANOVA with the within-subject RM factor of treatment (pre-AngII, post-AngII) and the between-subjects factors of sex (male, female) and prenatal exposure (sham-exposed, METH-exposed). ANOVAs were followed by Holm-Sidak multiple-comparison tests, as appropriate.

All analyses were performed using GraphPad 10.5.0 with the significance level set at *p* < 0.05.

## 3 Results

### 3.1 METH-induced locomotor activity

**FIGURE 2:**
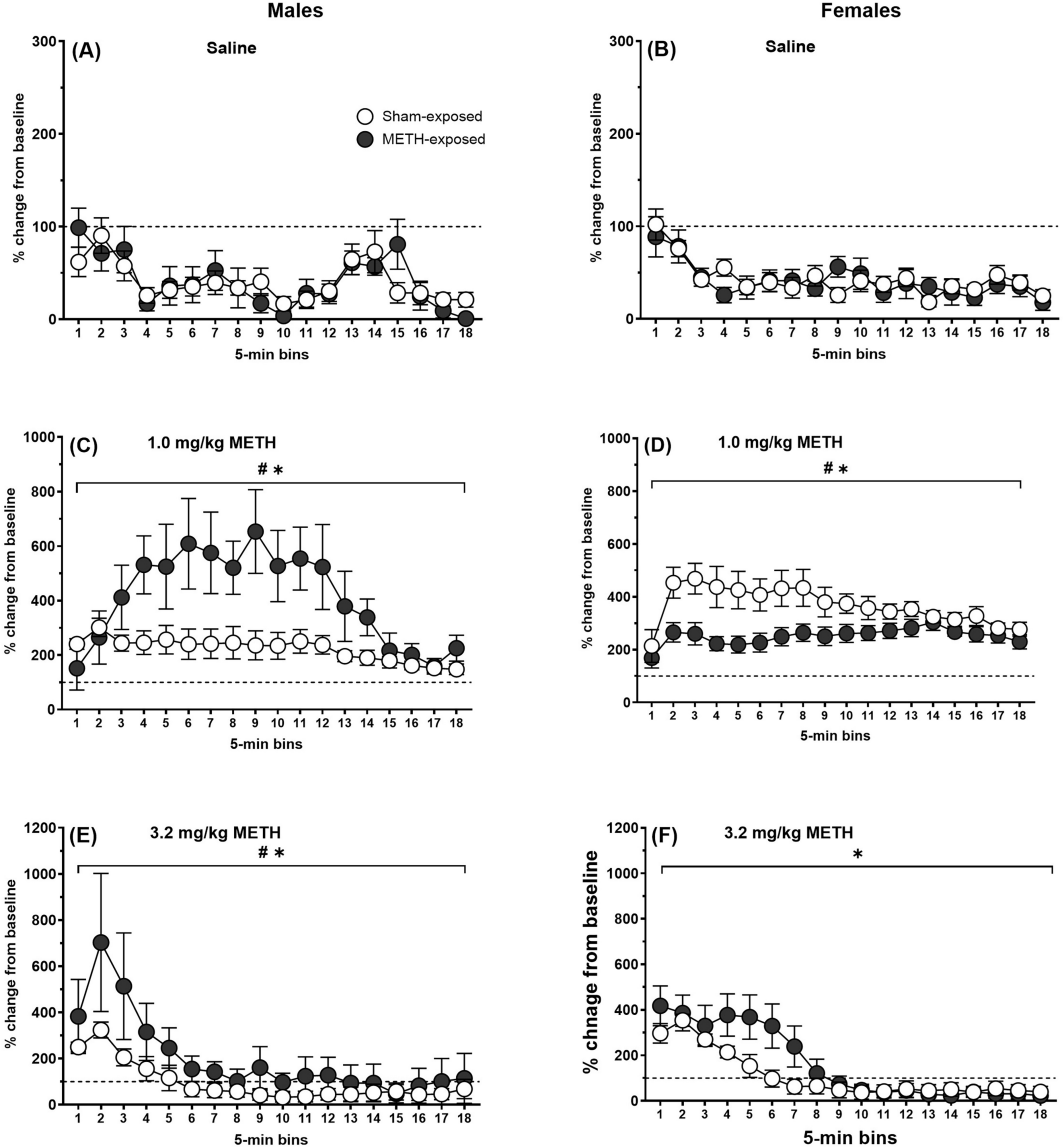
METH-induced locomotor activity in male (left panel; Sham-exposed *n* = 8, METH-exposed *n* = 4) and female (right panel; Sham-exposed *n* = 7, METH-exposed *n* = 6) aged Sham-exposed and METH-exposed offspring. Data are shown as percent change from baseline (dashed line) within 5 min bins (Mean ± S.E.M.) after *s.c*. METH administration. Upper panel shows data after saline administration in males **(A)** and females **(B).** The middle panel shows data after 1.0 mg/kg METH administration in males **(C)** and females **(D).** The bottom panel shows data after 3.2 mg/kg METH administration in males **(E)** and females **(F)**. #Indicates a main effect of prenatal exposure, *Iindicates a prenatal exposure x time bin interaction.

As depicted in Figure 2, top panel, locomotor activity after a saline injection decreased across time in both sexes, as indicated by a significant main effect of time bins in males [*F*(17, 170) = 5.01, *p* < 0.001, Figure 2A] and females [*F*(17, 187) = 6.42, *p* < 0.001, Figure 2B]. There were no significant main effects or interactions of prenatal exposure in either sex (*p* > 0.52).

Locomotor activity after 1.0 mg/kg METH showed a distinct sex-specific pattern (Figure 2, middle panel). Male rats of both exposure groups showed a time-dependent increase in locomotor activity, as indicated by a main effect of time bins [*F*(17, 170) = 7.71, *p* < 0.001]. As depicted in Figure 2C, METH-exposed males showed a higher increase in locomotor activity after the METH injection. They maintained a higher overall level of activity throughout the session than Sham-exposed males. These observations were reflected by a main effect of prenatal exposure [*F*(1, 10) = 8.10, *p* < 0.02] and a prenatal exposure x time bins interaction [*F*(17, 170) = 4.58, *p* < 0.001].

As in males, female rats of both exposure groups showed time-dependent increases in locomotor activity, as indicated by a main effect of time bins [*F*(17, 187) = 3.79, *p* < 0.001]. As depicted in Figure 2D, METH-exposed females showed a blunted increase in locomotor activity after the METH injection and maintained an overall lower level of activity throughout the session, compared to Sham-exposed females. These observations were reflected by a main effect of prenatal exposure [*F*(1, 11) = 5.99, *p* < 0.04] and a prenatal exposure x time bins interaction [*F*(17, 187) = 2.71, *p* < 0.001].

Locomotor activity in response to 3.2 mg/kg METH administration showed a similar pattern in males and females (Figure 2, bottom panel), with an initial increase in locomotor activity, followed by a marked reduction in activity, indicative of the occurrence of METH-specific stereotypic behavior (i.e., rearing), as indicated by a significant main effect of time bin in both sexes [males: *F*(17, 170) = 15.35, *p* < 0.001; females: *F*(17, 187) = 22.96, *p* < 0.001]. As depicted in Figure 2E, METH-exposed males showed a larger initial increase in locomotor activity and a blunted drop in activity later in the session compared to Sham-exposed males, as indicated by a significant prenatal exposure x time bins interaction [*F*(17, 170) = 2.19, *p* < 0.006]. As depicted in Figure 2F, METH-exposed females showed a larger initial increase in locomotor activity and a blunted drop in activity later in the session compared to Sham-exposed females, as indicated by a significant prenatal exposure x time bins interaction [*F*(17, 187) = 2.75, *p* < 0.001].

### 3.2 Mean, diastolic, and systolic blood pressure

**FIGURE 3:**
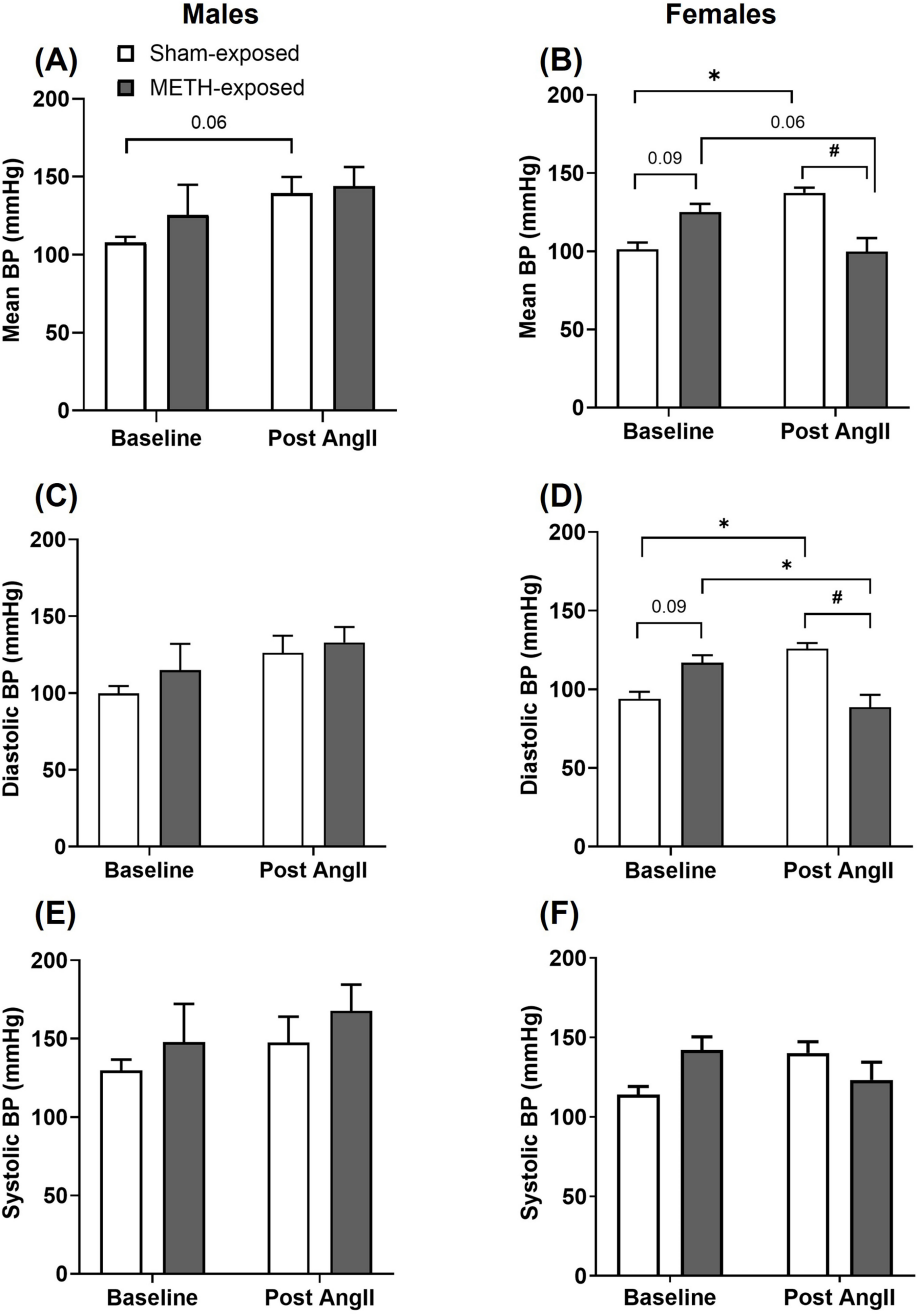
Baseline blood pressure and after 10d AngII treatment in male (left panel; Sham-exposed *n* = 5, METH-exposed *n* = 4) and female (right panel; Sham-exposed *n* = 10, METH-exposed *n* = 8) aged Sham-exposed and METH-exposed offspring. Data are shown as mmHg (Mean ± S.E.M.). Upper panel shows mean blood pressure in males **(A)** and females **(B).** The middle panel shows diastolic blood pressure in males **(C)** and females **(D).** The bottom panel shows systolic blood pressure in males **(E)** and females **(F)**. #Indicates a main effect of prenatal exposure, *Indicates a significant difference between baseline and post AngII.

For mean blood pressure, females exhibited lower blood pressure compared to males (main effect of sex [*F*(1, 23) = 4.56, *p* < 0.05]), and AngII treatment generally induced hypertension (main effect of treatment [*F*(1, 23) = 9.55, *p* < 0.01]), as revealed by the 3-way ANOVA. In addition, there were significant interactions of treatment x prenatal exposure [*F*(1, 23) = 14.25, *p* < 0.001], treatment x sex x prenatal exposure [*F*(1, 23) = 5.98, *p* < 0.03], and a trend to a sex x treatment interaction [*F*(1,23) = 3.95, *p* < 0.06]. Holm-Sidak *post-hoc* analysis revealed the following sex-specific exposure-dependent differences: In male Sham offspring, AngII showed a trend to induce hypertension (*p* < 0.06), an effect that was not observed in METH-exposed male offspring (Figure 3A). As depicted in Figure 3B, METH-exposed females showed a tendency for increased baseline blood pressure compared to Sham-exposed females (*p* < 0.09). AngII induced significantly elevated mean blood pressure in Sham-exposed females (*p* < 0.001) and a trend to reduced blood pressure in METH-exposed females (*p* < 0.06). In addition, METH-exposed females showed lower mean blood pressure after AngII compared to Sham-exposed females (*p* < 0.002).

Similarly to mean blood pressure, females overall exhibited lower diastolic blood pressure compared to males (main effect of sex [*F*(1, 23) = 5.02, *p* < 0.04]) and AngII treatment induced elevated diastolic blood pressure (main effects of treatment [*F*(1, 23) = 5.51, *p* < 0.03]). There were significant interactions of treatment x exposure [*F*(1, 23) = 11.14, *p* < 0.003] and sex x treatment x exposure [*F*(1, 23) = 6.34, *p* < 0.02]. *Post-hoc* analysis revealed the following sex-specific and exposure-dependent measures: There were no significant differences between METH-exposed and sham male offspring (Figure 3C). In sham female offspring, AngII treatment led to a significant increase in diastolic blood pressure compared to baseline (*p* < 0.006). In contrast, it induced a reduction in METH-exposed females (*p* < 0.04), as well as a reduction compared to sham-exposed AngII-treated females (*p* < 0.001, Figure 3D).

There were no significant effects for systolic blood pressure. However, males tended to have higher systolic blood pressure than females (*p* < 0.07; Figures 3E, F).

## 4 Discussion

The current study assessed the impact of prenatal METH exposure on potential sex differences in METH sensitivity and susceptibility to cardiovascular problems in aged offspring. To determine METH sensitivity, METH-induced hyperlocomotion was assessed compared to sham-exposed control offspring. METH administration induced dose-dependent activity in all offspring, with METH-exposed offspring showing a dose-dependent sex-specific sensitivity to METH, with males exhibiting an increased activity after a medium METH dose, whereas females showed decreased activity at the same dose. Both male and female METH-exposed offspring showed increased activity compared to controls at a high METH dose, which induced stereotypic behavior (i.e., rearing). When assessing the impact of prenatal METH exposure on cardiovascular function in aged offspring, we observed sex-specific effects compared to sham control offspring. While there were no prenatal-exposure-dependent effects in males, female METH-exposed offspring showed a tendency toward elevated baseline blood pressure and reduced blood pressure after AngII compared with sham-exposed offspring.

Spontaneous locomotor activity and activity after a mild stressor (i.e., saline injection) were not different between the prenatal exposure groups. This is in line with previous work showing that prenatal METH exposure, either via non-contingent injections or maternal self-administration under limited-access conditions, did not impair exploratory behavior under no- or low-stress conditions (Schutová et al., 2010; Hrubá et al., 2012; Pareek et al., 2023). Administration of acute METH has been shown to dose-dependently induce hyperlocomotion, with high doses initially reducing locomotor activity due to the occurrence of stereotypic behavior (Bhimani et al., 2021; Milesi-Hallé et al., 2007). Prenatal METH-exposure has been shown to affect dose-dependent locomotor activity in a sex-dependent manner, with males being more sensitive to the effects than females, particularly when intermediate doses of METH were administered (Bubenikova-Valesova et al., 2009; Pareek et al., 2023). In our current study, administration of 1.0 mg/kg METH induced sustained increased locomotor activity in METH-exposed males, compared to sham-exposed male offspring. The pattern of increased METH-induced hyperlocomotion resembles that observed after behavioral sensitization. Behavioral sensitization describes the phenomenon where repeated METH administrations induce an increased response to a challenge dose of METH, compared to animals that did not receive repeated METH administrations (McDaid et al., 2006; Wearne et al., 2017). Our results in males replicated those of Pareek et al. (2023), where dams of METH-exposed offspring had access to METH self-administration under limited-access conditions throughout pregnancy. In the Pareek study, female offspring under the same limited-access conditions did not differ from their control counterparts. This contrasts with our current study, in which METH-exposed female offspring exhibited reduced locomotor activity compared with their sham-exposed controls. This difference in findings could be attributed to differences in the subjects’ ages or to differences in the level of prenatal METH exposure between the two studies. While the Pareek study tested adult rats, the subjects in our study were significantly older, and the females had already stopped cycling. While studies investigating METH interactions in aged rats of both sexes are lacking, there is a clear link between METH sensitivity, age, and ovarian sex hormones (Arunogiri et al., 2021; Bishnoi et al., 2021; Slamberová et al., 2013). In addition, dams in the Pareek study self-administered METH under limited-access conditions, which resulted in a moderate daily intake across gestation (2.5 mg/kg/day; Rüedi-Bettschen and Platt, 2017). In our current study, dams had extended access to METH self-administration, resulting in substantial daily METH intake throughout gestation (5 mg/kg/day). Therefore, the higher exposure may be a contributing factor, as dose-dependent effects have been reported (Acuff-Smith et al., 1996).

In response to a higher dose of METH (3.2 mg/kg), all offspring exhibited an initial surge in activity, followed by a pronounced drop in locomotor activity and a simultaneous emergence of stereotypic behavior (i.e., repetitive rearing). Yet METH-exposed offspring of both sexes exhibited a temporary resistance to stereotypic behavior compared to sham offspring. These findings support previous studies that observed less pronounced hypolocomotion and decreased rearing in offspring prenatally exposed to stimulants compared to controls after a high acute dose of METH (Ruda-Kucerova et al., 2018; Schutová et al., 2010). Taken together, these findings suggest that prenatal METH exposure affects METH-induced activity, in terms of locomotion and severity of stereotypic behavior, and that the effects are sex dependent. In contrast to studies in adult METH-exposed offspring, where males are usually more sensitive to the stimulatory effects than females (Schutová et al., 2013), in aged offspring, females are the more profoundly affected sex. Further studies are needed to elucidate the mechanisms, including the hormonal milieu, contributing to the age-dependent, long-term effects of prenatal METH exposure.

When METH-exposed and sham control offspring were assessed for susceptibility to changes in cardiovascular function, there was a clear sex-dependent effect. At baseline, METH-exposed offspring of both sexes tended to have higher blood pressure compared to sham offspring. Hypertension has been shown as a result of acute and prolonged METH use in adults (Rusyniak et al., 2012; Vaupel et al., 2016), but only a few studies are elucidating the impact of prenatal METH exposure on the susceptibility to cardiovascular problems in adult offspring. In these studies, prenatal METH exposure had no impact on baseline systolic and diastolic blood pressure in adult male or female offspring (Chavva et al., 2022; Harrison et al., 2021). Our results confirm these findings regarding systolic and, in part, diastolic blood pressure. However, while our results for diastolic blood pressure in females are not statistically significant, the trend to higher blood pressure in METH-exposed offspring is substantial, and our results thus do not fully align with the reported findings. One main difference between these and our study is the way prenatal METH exposure is achieved. In the other studies, METH is administered non-contingently only throughout pregnancy, whereas in our study, dams self-administer METH prior to and throughout pregnancy. Therefore, we expect physiological changes in our dams to happen even before conception. In addition, we tested our offspring at a significantly later time point, which could also influence the emergence of baseline blood pressure differences. One could also argue that prenatal METH exposure increases stress reactivity, and therefore, the increases in blood pressure are simply a reflection of stress reactivity. While this is a possibility that would require further evaluation with stress hormone detection during the tail-cuff blood pressure assessment, we do not believe this is the case. In preparation for blood pressure assessment, rats were habituated extensively to the apparatus and the cuffs, and testing was conducted only when a rat was visibly relaxed (e.g., normal breathing, relaxed tail) after at least 2 habituation sessions.

When assessing the effect of AngII treatment on METH- and sham-exposed offspring, we observed sex-dependent effects. While AngII treatment induced similar effects in male offspring (i.e., a slight increase in mean blood pressure) in both exposure groups, female offspring demonstrated clear prenatal exposure-dependent effects. In sham-exposed females, AngII induced a significant increase in mean blood pressure. In METH-exposed females, however, AngII treatment decreased mean and diastolic blood pressure compared to baseline. The current study is the first to use AngII treatment to evaluate cardiovascular function in aged offspring prenatally exposed to METH. Other studies, investigating the impact of prenatal stimulant exposure on AngII sensitivity, have identified a sensitized response to AngII in adult males but not in females (Xiao et al., 2008; Yu et al., 2017). The discrepancy in findings could stem from several factors. First, the stimulant studied was nicotine, which may affect the results of the study. Secondly, we employed a 10-day steady-state infusion of AngII before testing, whereas the other studies administered a single bolus dose of AngII before blood pressure assessment. Lastly, the subjects in our study were significantly older than those in the other study. Rats in menopause/andropause have been shown to exhibit hypertension over time, and males were more sensitive to the development of hypertension compared to females (Perusquía and Herrera, 2025). However, chronic METH use has been shown to disrupt the hypothalamic-pituitary-ovarian axis, and it has been speculated that prenatal METH exposure may affect estrogen secretion in female offspring, thereby compromising its protective effects on the cardiovascular system (Shen et al., 2014), making female METH-exposed offspring particularly vulnerable to cardiovascular insults. For example, it was reported that METH-exposed adult female offspring had elevated end-diastolic pressure during recovery from ischemia and higher sensitivity to myocardial ischemic injury compared to males (Rorabaugh et al., 2016). There are marked sex differences in the effects of the renin-angiotensin system on cardiovascular function and in the differential expression of AngII receptors, which are sensitive to METH administration (Medina et al., 2020; Zhao et al., 2021). Therefore, it is possible that AngII receptor expression could be altered by prenatal METH exposure, further impacting whether AngII would induce hypertension in aged offspring (Davisson et al., 2000). Future studies are needed to explore how AngII receptor expression in METH-exposed offspring influences blood pressure regulation and responses to Ang-II-induced hypertension.

The current study provides important insights into how prenatal METH exposure influences sensitivity to subsequent METH challenges and susceptibility to cardiovascular dysfunction in aged offspring. Nonetheless, several limitations should be considered. First, male sample sizes were smaller than those of females in both behavioral and cardiovascular assessments. Although male and female siblings were used, age-related attrition was more pronounced in males. To ensure the reproducibility of our findings, we restricted analyses to animals that completed the full protocol and showed no signs of declining health, evidenced by stable body weight throughout the experiment. Second, blood pressure was assessed using the tail-cuff method rather than direct arterial catheterization. Given the animals’ advanced age, we prioritized the least invasive approach; however, this method is associated with greater variability and potential confounding factors, such as stress. Future work should determine whether age and hormonal status interact with prenatal METH exposure to shape cardiovascular outcomes. Longitudinal studies using implantable telemetry devices to measure arterial pressure in freely moving animals would provide more precise and continuous cardiovascular data. Additionally, future studies should include the collection of tissues and blood vessels to begin identifying the mechanistic pathways underlying the observed effects.

In conclusion, the results of the current study indicate that prenatal METH exposure, achieved via maternal self-administration under extended-access conditions, leads to sex-dependent alterations in the sensitivity to the locomotor stimulatory effects of METH and in blood pressure in aged offspring. Interestingly, as male offspring have been repeatedly shown to be less resilient to changes in the pre- and postnatal environment as adults (Pareek et al., 2023; Pryce et al., 2001; Pryce et al., 2003; Rüedi-Bettschen and Platt, 2017), in our current study, females are more profoundly affected by prenatal METH exposure. This highlights the importance of studying aged offspring to determine sex-specific risks during andropause/menopause.

## Data Availability

The raw data supporting the conclusions of this article will be made available by the authors, without undue reservation.
